# The Function of Gas Vesicles in Halophilic Archaeaand Bacteria: Theories and Experimental Evidence

**DOI:** 10.3390/life3010001

**Published:** 2012-12-27

**Authors:** Aharon Oren

**Affiliations:** Department of Plant and Environmental Sciences, The Alexander Silberman Institute of Life Sciences, The Hebrew University of Jerusalem, 91904, Jerusalem, Israel; E-Mail: aharon.oren@mail.huji.ac.il; Tel.: +972-2-6584951; Fax: +972-2-6584425

**Keywords:** gas vesicles, *Halobacterium*, *Haloferax*, *Haloquadratum*, *Haloplanus*, *Halogeometricum*, bacteriorhodopsin, oxygen

## Abstract

A few extremely halophilic Archaea (*Halobacterium salinarum*, *Haloquadratum walsbyi*, *Haloferax mediterranei*, *Halorubrum vacuolatum*, *Halogeometricum borinquense*, *Haloplanus* spp.) possess gas vesicles that bestow buoyancy on the cells. Gas vesicles are also produced by the anaerobic endospore-forming halophilic Bacteria *Sporohalobacter lortetii* and *Orenia sivashensis*. We have extensive information on the properties of gas vesicles in *Hbt. salinarum* and *Hfx. mediterranei* and the regulation of their formation. Different functions were suggested for gas vesicle synthesis: buoying cells towards oxygen-rich surface layers in hypersaline water bodies to prevent oxygen limitation, reaching higher light intensities for the light-driven proton pump bacteriorhodopsin, positioning the cells optimally for light absorption, light shielding, reducing the cytoplasmic volume leading to a higher surface-area-to-volume ratio (for the Archaea) and dispersal of endospores (for the anaerobic spore-forming Bacteria). Except for *Hqr. walsbyi* which abounds in saltern crystallizer brines, gas-vacuolate halophiles are not among the dominant life forms in hypersaline environments. There only has been little research on gas vesicles in natural communities of halophilic microorganisms, and the few existing studies failed to provide clear evidence for their possible function. This paper summarizes the current status of the different theories why gas vesicles may provide a selective advantage to some halophilic microorganisms.

## 1. Introduction

A small number of species of extremely halophilic Archaea of the family *Halobacteriaceae* (8 out of the 137 species with names with standing in the nomenclature as of August 2012) [1] are able to produce gas vesicles. These include two organisms that have been investigated in-depth in the past decades as models for the study of gas vesicle production: *Halobacterium salinarum* and *Haloferax mediterranei*. These also include the intriguing flat, square or rectangular *Haloquadratum walsbyi*, which is abundantly found in hypersaline brines e.g. in saltern crystallizer ponds worldwide. Such cells are buoyant: liquid cultures of *Hbt. salinarum* left standing form a pellicle at the surface.

Gas vesicle production is by no means restricted to the *Halobacteriaceae*. Many representatives of the bacteria belonging to different classes possess them. The ability to form gas vesicles is especially widespread among the cyanobacteria. Gas vesicles often occur in clusters ('gas vacuoles') visible as bright refractile bodies in the phase contrast microscope. Masses of gas-vacuolate cyanobacteria are often found floating on the surface of freshwater lakes, buoyed up due to the high content of gas vesicles that bestow buoyancy on the cells. There are also a few methanogenic Archaea that produce gas vesicles.

Presence of refractile 'gas vacuoles' in 'Bacterium halobium' isolated from salted herring, a strain of the species now known as *Hbt. salinarum*, was first reported by Helena Petter in the early 1930s [2,3]. She grew red colonies from salted herring and dried cod: three isolates formed transparent colonies; four had opaque colonies with gas vesicles. She also published the first drawings of *Halobacterium* cells with gas vacuoles. Already then she suggested that the presence of gas vesicles and the buoyancy these vesicles bestow on the cells can be of considerable ecological advantage: the vesicles may buoy the cells to the surface of brine pools and salt lakes, where they would benefit from higher concentrations of oxygen. Oxygen and other gases are much less soluble in saturated salt solutions than in freshwater or in seawater. For example, distilled water at 20 °C in equilibrium with air contains 9.10 mg/L (284 μM) O_2_, while at 260 ppt (parts per thousand; g/kg of solution) salinity there is only 1.67 mg/L (52 μM). At 35 °C the values are reduced to 6.92 mg/L (216 μM) and 1.51 mg/L (47 μM), respectively [4]. Therefore oxygen might become a limiting factor at high salt. This interpretation was quickly adopted by others. Trijntje Hof described in 1935 a similar gas-vacuolate strain [5]. From those days onward, *Halobacterium* became a popular object for the study of gas vesicles, as shown by the admirable electron micrographs—even including early stereopictures of surprisingly high quality—published by Houwink in 1956 [6] and by the early physiological studies by Helge Larsen and his coworkers [7].

Since those early times many possible functions have been suggested for gas vesicle synthesis in different members of the *Halobacteriaceae*: buoying the cells towards more oxygen-rich surface layers in hypersaline water bodies to prevent oxygen limitation, reaching higher light intensities for thelight-driven proton pump bacteriorhodopsin, positioning the cells in an optimal orientation for light absorption, light shielding, and reducing the cytoplasmic volume leading to a higher surface-area-to-volume ratio. These conclusions were mainly based on laboratory studies, and very few investigations on gas vesicles were performed in the field in natural communities of halophilic microorganisms. In this paper I will attempt to evaluate the relative merit of the different theories proposed to explain why gas vesicles may provide a selective advantage to some halophilic microorganisms.

## 2. What Gas Vesicles Are

Gas vesicles are hollow cylindrical or spindle-shaped structures, built of protein subunits. In the *Halobacteriaceae* the size of the vesicles varies between 0.2–1.5 μM in length and they are ~0.2 μM in diameter. A cell of *Hbt. salinarum* can contain up to 70 spindle-shaped gas vesicles when grown aerobically at 40 °C [8]. The wall of the vesicles consists of a single layer of the 7–8 kDa GvpA protein, which forms 4.6 nm wide ‘ribs’ that run nearly perpendicular to the long axis of the vesicle. A second structural component, the 31–42 kDa GvpC, is a protein that contains internal repeats. It strengthens the vesicle by attaching to its outer surface [8,9]. The majority of the gas vesicles in *Hbt. salinarum* have the form of a wide, rounded bicone (‘lemon-shaped’ or ‘spindle-shaped’). The same organism also produces some longer, narrower and cylindrical gas vesicles [8]. 

The GvpA protein has a very high content of hydrophobic amino acids, and is highly conserved in all prokaryotes that produce gas vesicles, Archaea as well as Bacteria [9]. The GvpA protein is one of the few proteins in halophilic Archaea that do not require salt for stabilization. The GvpC protein strengthens the structure of the vesicles, assists in their assembly, and to a large extent determines the shape of the gas vesicles. 

Similar to the gas vesicles of other prokaryotes, the vesicles found in the members of the *Halobacteriaceae* are sensitive to pressure. They are typically weaker than the gas vesicles of cyanobacteria which generally withstand pressures of up to 0.2–0.3 MPa (~2–3 atmospheres): a pressure of 0.09 MPa causes collapse of half of the gas vesicles in *Hbt. salinarum*, while the weakest gas vesicles within the cells are already destroyed by a pressure of 0.05 MPa [9,10]. These critical collapse pressures set a limit to the depth in a water column in which gas-vacuolate cells can occur. A pressure of 0.09 MPa corresponds to a water depth of ~9 m in fresh water or ~7.3 m in salt-saturated brine. Part of the variation in critical pressure can be explained by the variation in cylinder radius of the gas vesicles. The density of gas vesicles in different prokaryotes was estimated to vary from 60 kg m^−3^ for the widest vesicles to 210 kg m^−3 ^for the narrowest ones, present in the marinecyanobacterium *Trichodesmium* [9].

In-depth studies on the genes involved in gas vesicle production and their regulation were thus far performed only with *Hbt. salinarum* and *Hfx. mediterranei*. In both species gas vesicle production increases in the stationary phase, and the ability to produce gas vesicles is easily lost by mutation [8,11,12]. Formation of gas vesicles requires 8–14 different proteins, including the two structural proteins GvpA and GvpC [13,14,15]. The product of the gene *gvpD* is negative regulator. Transformation of *Hfx. volcanii* with an *mc-vac* construct containing a *gvpD* deletion leads to cells with high numbers of gas vesicles [16]. Further information about the (putative) functions of the different genes involved can be found in a number of papers [17,18,19], including a recent review article [8]. How environmental factors such as oxygen and salt concentration that affect gas vesicle biosynthesis are transduced to the regulators and influence transcription is largely unknown.

## 3. How Common Are Gas Vesicles Among the Species of *Halobacteriaceae*?

The ability to produce gas vesicles is not widely distributed among the halophilic Archaea. Out of the 40 genera and 137 species of *Halobacteriaceae* (as of August 2012) with names with standing in the nomenclature [1], no more than eight species belonging to six genera were reported to possess gas vesicles (Table 1). 

**Table 1:** Gas vesicle producing halophilic Archaea (family *Halobacteriaceae*).

Genus	Species	Source of isolation	Flagellar motility	Bacteriorhodopsin / halorhodopsin	References
*Halobacterium*	*Hbt. salinarum*	Salted fish	+	+	[1]
*Haloferax*	*Hfx. mediterranei* (basonym: *Halobacterium mediterranei* )	Saltern, Spain	weak	- ^a^	[20]
*Halogeometricum*	*Hgm. borinquense*	Saltern, Puerto Rico	-	- ^a^	[21]
*Haloplanus*	*Hpl. natans*	Experimental outdoor pond, Dead Sea, Israel	-	NR	[22]
	*Hpl. vescus*	Saltern, China	+	NR	[23]
	*Hpl. aerogenes*	Saltern, China	+	NR	[24]
*Haloquadratum*	*Hqr. walsbyi*	Salterns, Australia and Spain	-	+	[25]
*Halorubrum*	*Hrr. vacuolatum* (basonym: *Natronobacterium vacuolatum* corrig.)	Lake Magadi, Kenya	-	NR	[26]

^a^ as deduced from the genome sequence; NR = not reported.

Gas-vacuolate *Halobacterium* strains have been isolated many times, and these include the isolates from salted fish used in the early studies by Petter, Houwink, Larsen, and others [1,2,3,5,6,7]. All known gas-vacuolate strains of *Halobacterium* can be assigned to the species *Hbt. salinarum*, including the widely studied strain NRC-1 [27]. The other species of the genus, *Hbt. jilantaiense* retrieved from a salt lake in Inner Mongolia, China, *Hbt. noricense* from a salt mine and ‘*Hbt. piscisalsi*’ from fermented fish in Thailand (later described as a junior synonym of *Hbt. salinarum*) lack the property.

Out of the 11 currently recognized species within the genus *Haloferax*, *Hfx. mediterranei* is the only one with gas vesicles. It was isolated from an enrichment culture for extreme halophiles able to grow on single carbon sources, using brine from a Spanish saltern pond as inoculum [20]. 

The genus *Halogeometricum* contains the gas-vacuolate *Hgm. borinquense* from a saltern pond in Puerto Rico [21]. No further studies on its gas vesicles were reported. The description of the second species of the genus, *Hgm. rufum*, did not mention the presence of gas vesicles.

**Figure 1:**
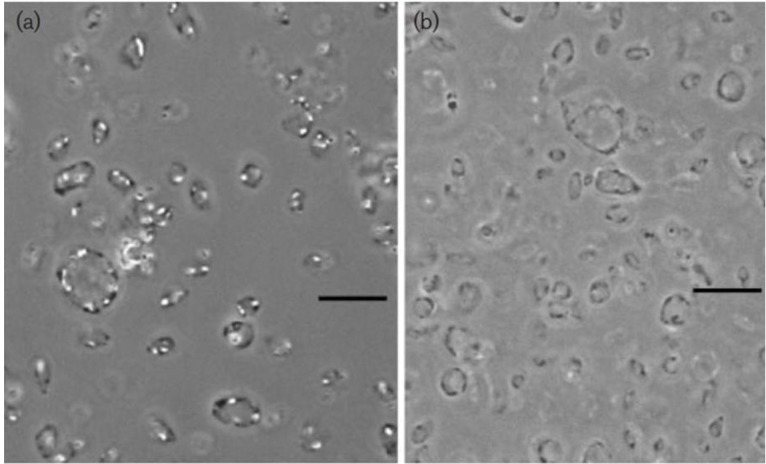
Phase-contrast micrograph of gas-vacuolate cells of *Haloplanus natans* strain RE-101^T^ (**a**) and cells after centrifugation, causing collapse of the gas vesicles (**b**) Bars, 10μM. Note the concentrations of gas vesicles at the periphery of the cells in panel (**a**). From EleviBardavid *et al.*, 1997 [22], reproduced with permission from the Society for General Microbiology, Reading, UK.

All three described species of the genus *Haloplanus* are gas-vacuolate: *Hpl. aerogenes*, *Hpl. natans* (Figure 1), and *Hpl. vescus. Hpl. aerogenes* and *Hpl. vescus* were isolated from solar salterns [23,24]; *Hpl. natans* was obtained from outdoor simulation ponds in which mixtures of Dead Sea and Red Sea waters were incubated [22].

**Figure 2:**
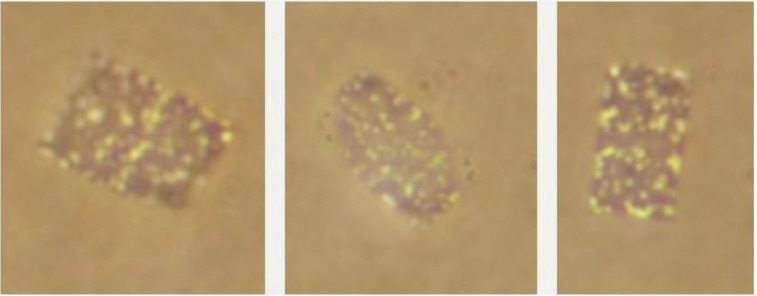
Flat, square to rectangular, *Haloquadratum walsbyi*-type cells from the saltern crystallizer ponds of Eilat, Israel. Note the division plane in the left panel. Photograph: O. Shapiro, N.Siboni and N.Pri-El.

The genus *Haloquadratum* consists of a single species, *Hqr. walsbyi*. This is the flat, square-rectangular archaeon first observed by Walsby in a brine pool on the Sinai peninsula, Egypt [28], and only isolated more than two decades later from saltern crystallizer ponds [25,29,30,31]. *Hqr. walsbyi* is probably the only gas-vacuolate halophilic archaeon that is abundantly found in hypersaline brines worldwide. Figure 2 shows a picture of such square gas-vacuolate cells in the crystallizer brine of a saltern in Israel. Generally the gas vesicles are not distributed evenly throughout the cells, but they are concentrated near the edges of the squares. This can be seen in the left panel of Figure 2 and in many published micrographs and electron micrographs [30,31,32,33].

*Halorubrum vacuolatum* from Lake Magadi, Kenya [26], an alkaliphilic species of small cells originally described as *Natronobacterium vacuolatum* (*vacuolata*), is the only of the 25 species of the genus *Halorubrum* that carries gas vesicles. Its cells are very small, ~1–1.5 μM in the stationary phase. Little information is available on the properties of its gas vesicles and on the regulation of their production. It lacks gas vesicles when grown in salt concentrations below 15% [18,34].

Some gas-vacuolate species are actively motile by means of flagella, so that their mode of positioning in the water column may be determined both by their buoyancy due to the gas vesicles and by flagella-driven motility. Calculations have shown that, at least in slow growing cells, upward movement mediated by gas vesicles is energetically less expensive than by rotating flagella [9,35]. This does not imply that passive movement mediated by gas vesicles is not energetically costly. To produce sufficient gas vesicles so that 10% of the cell volume is occupied by gas, an equivalent of ~7.2% of the total protein synthesized or ~4.3% of the dry cell mass must be gas vesicle protein (values determined for the cyanobacterium *Anabaena*) [9]. To justify this cost there must be some selective advantage to the production of gas vesicles.

## 4. The Possible Advantages of Gas Vesicles to Halophilic Archaea: Laboratory Studies

To elucidate the possible selective advantages of the production of gas vesicles by members of the *Halobacteriaceae*, laboratory studies have been performed mostly with gas-vacuolate *Halobacterium* strains, with *Hfx. mediterranei*, or with transformants of *Hfx. volcanii* (a non-vacuolate species) carrying genes for gas vesicle production derived from *Hfx. mediterranei*.

### 4.1. Competition for Limiting Oxygen

The idea that gas vesicles may buoy cells to the surface of brine pools and salt lakes where they would benefit from higher concentrations of oxygen was already proposed by Petter in the 1930s [2,3]. The ability to float to the brine surface may be advantageous for an aerobic halophilic microorganism as the solubility of oxygen and other gases in salt-saturated brines is low. To what extent oxygen is indeed a limiting factor in those environments inhabited by gas-vacuolate members of the *Halobacteriaceae* will be discussed in Section 5.2. 

To experimentally test whether under oxygen limitation there may be a selective advantage to gas-vacuolate types, competition experiments were set up between a gas-vacuolate strain of *Hbt. salinarum* (strain PHH1) and a mutant affected in gas vesicle synthesis [36]. In shaken cultures both strains grew equally well, but in deep static cultures, where, due to the community respiration, steep vertical oxygen concentration gradients were established, cells of the wild type floated and became dominant, probably due to their successful competition for oxygen which was in short supply. In shallow static cultures, however, the gas-vesicle deficient mutant won the competition. A possible explanation is that under the conditions employed the wild type wasted much energy to produce unnecessary gas vesicles, while the mutant had a lower protein burden.

### 4.2. Can Gas Vesicles Function as Intracellular Oxygen Reservoirs?

The O_2_ carrying capacity of water at 37 °C is ~9.7 mol m^−3^ MPa^−1^. For gas vesicles the value is 388 mol m^−3^ MPa^−1^, *i.e.*, 40-times as large. Therefore the use of isolated gas vesicles (from the cyanobacterium *Anabaena flos-aquae*) was explored as oxygen carriers to increase the oxygen supply in mammalian cell culture systems [37]. Addition of 1% volume of gas vesicles thus leads to a 39% increase in oxygen carrying capacity, a 10% gas vesicle volume results in a 390% increase. The question can therefore be asked whether gas vesicles inside halophilic Archaea may serve as intracellular oxygen reservoirs. The answer probably must be negative. The gas vesicle wall and the cell membrane are both highly permeable to gases, so gases will rapidly equilibrate between the cell and the surrounding brine. Therefore the cell cannot “store” oxygen to keep it for its own use, and the oxygen content of the gas vesicles will rapidly decrease when oxygen is consumed by community respiration. Even in dense communities of halophilic Archaea the total volume occupied by the gas vesicles is still small. For example, in a hypothetical case in which a brine is inhabited by 10^8^ *Hqr. walsbyi* cells that are 3 × 3 × 0.2 μM in size and have 20% of their intracellular volume occupied by gas vesicles, the total O_2_ carrying capacity of the brine will be increased only by ~0.7% due to the presence of the gas vesicles.

Diffusion of oxygen in air is 5,700 times (at 20 °C) to 20,000 times (40 °C) more rapid than in water [38]. This may mean that oxygen present in the gas vesicles may be made accessible much more rapidly than oxygen dissolved in the cytoplasm or in the surrounding brine. Whether this is indeed of physiological importance for these small cells, in which the relevant diffusion distances are less than 1 μM, may be doubted. 

### 4.3. Is Gas Vesicle Biosynthesis Induced by Anaerobiosis?

Although basically an organism with an aerobic life style, *Hbt. salinarum* possesses different modes of anaerobic growth. These include anaerobic respiration using dimethylsulfoxide (DMSO) or trimethylamine *N*-oxide (TMAO) as the electron acceptor [39] and fermentative growth on arginine [40,41]. Arginine fermentation is not commonly found among the *Halobacteriaceae*, and appears to be a special feature of the genus *Halobacterium*. Therefore a specific enrichment procedure could be developed for this genus, based on its ability to grow anaerobically in arginine-containing media [42].

In contrast to what might be expected, anaerobiosis represses the formation of gas vesicles. When grown or incubated under anoxic conditions, three strains of *Hbt. salinarum*, *Hfx. mediterranei* and *Hfx. volcanii* transformants lacked gas vesicles altogether, or had a much lower content than aerobically grown cells. Even the gas vesicle-overproducing Δ*gvpD* transformants did not produce gas vesicles under anaerobic conditions, demonstrating that the repressing protein GvpD was not involved. Presence of large amounts of GvpA in the cells' cytoplasm implied that the assembly of gas vesicles was inhibited [43]. *Hbt. salinarum* cells grown by arginine fermentation contain only a few groups of tiny gas vesicles [43], whereas cells that grow using TMAO respiration contain few larger vesicles [44]. This may explain why in the anaerobic enrichment cultures for *Halobacterium* using arginine as energy source, gas vesicles were only seldom observed [42]; whether the enriched strains may have possessed the potential for gas vesicle production was not ascertained.

In oligonucleotide microarray studies with *Halobacterium* NRC-1, slight upregulation of *gvpA* and *gvpC* was reported following anaerobic incubation with arginine, while anaerobic cultures with TMAO showed apparent stronger (2–5-fold) upregulation of *gvpA*, *gvpC*, *gvpN*, and *gvpO*; these cells showed an abundance of gas vesicles [44,45]. This was probably due to the presence of citrate in the growth medium and not to the growth condition employed: citrate stimulates gas vesicle production. In a different medium lacking citrate, anaerobiosis strongly inhibited gas vesicle formation. Therefore only the gas vesicles already produced under oxic conditions might help the cells to avoid the anoxic zones of the brine [43].

### 4.4. Induction of Gas Vesicle Formation at High Salinity

*Hfx. mediterranei* is a versatile organism, able to grow over a wide range of salt concentrations. It produces gas vesicles only when the salt concentration in the medium exceeds 170 g/L [16,17,18,19,46,47]. The relative abundance of *mc-vac* mRNA in cells grown at 250 g/L salt was sevenfold higher than in cells grown in 150 g/L [16].

How the cells sense the salt concentration and transduce the information to regulate the transcription of gas vesicle genes is still unknown. The reason why high-salt-grown cells need more gas vesicles than low-salt-grown cells is also not clear, but it could be related to the lowered solubility of oxygen and other gases in concentrated brines. *Hbt. salinarum* has a much more restricted salinity range for growth, and therefore similar experiments with *Halobacterium* have not been reported. Effects of medium salinity on gas vesicle production were also examined in *Hrr. vacuolatum* [34].

### 4.5. Induction of Gas Vesicle Formation at Low Temperature

Different strains of *Hbt. salinarum* showed increased contents of gas vesicles when grown at a low temperature (15 °C). Growth is slow, and the cells formed are tightly filled with gas vesicles [48]. Oligonucleotide microarray experiments with *Halobacterium* NRC-1 showed a 1.8-fold to 8-fold increase in *gvp*ACNO expression and a 3-fold increase in expression of *gvp*DE in the cold [49].

As oxygen solubility in aqueous solutions is increased at lowered temperatures, the effect cannot be understood as an adaptation to an increased oxygen requirement. Growth and cell metabolism are also very slow at 15 °C, resulting in a low oxygen demand.

### 4.6. Are Gas Vesicles Formed to Increase Light Availability?

Some members of the *Halobacteriaceae* contain the membrane-bound light-driven proton pump bacteriorhodopsin and/or the light-driven chloride pump halorhodopsin. Such cells can directly convert light energy to a proton gradient (to drive generation of ATP, ion transport processes, *etc.*) or to pump chloride into the cells against the thermodynamic gradient. In some cases photoheterotrophic growth is even possible based on the energy of photons absorbed by bacteriorhodopsin [40,41,50]. Light and low oxygen concentrations are among the factors that trigger bacteriorhodopsin production with the formation of ‘purple membrane’ in *Hbt. salinarum*.

Some, but not all halophilic Archaea that produce gas vesicles also possess bacteriorhodopsin and halorhodopsin. Functional bacteriorhodopsin proton pumps are active in *Halobacterium* and in *Haloquadratum* (Table 1; see also [51]), but genes for bacteriorhodopsin or halorhodopsin were not detected in the genomes of *Hfx. mediterranei* and *Hgm. borinquense*; presence of the purple retinal pigments was never ascertained in *Haloplanus* spp. and in *Hrr. vacuolatum*.

Although upward flotation toward the brine surface may be beneficial for bacteriorhodopsin-containing cells to be able to harvest more light, a direct correlation between light intensity, presence or absence of bacteriorhodopsin, and gas vesicle production was never documented. These advantages could also be tested in competition experiments. This suggestion, made by Kessel *et al.* in 1985 [52], has not yet been followed up.

### 4.7. Are Gas Vesicles Formed as a Means of Protection Against Excess Light?

Another possible function was suggested for the gas vesicles, namely their use as light-shielding organelles to protect the organism exposed to high light intensities in its environment against harmful ultraviolet radiation. Light is scattered due to the large difference in refractive index between the gas vesicles and the cell's cytoplasm or the surrounding medium. This hypothesis was not supported by controlled laboratory experiments. No significant differences in sensitivity to UV radiation were observed between a gas-vacuolate *Halobacterium* strain and a gas-vesicle-deficient mutant. Therefore the gas vesicles are not effective as light-shielding organelles [53].

## 5. The Possible Advantages of Gas Vesicles to Halophilic Archaea: Field Studies

### 5.1. How Successful Are Gas-Vacuolate Species of Halobacteriaceae in Colonizing Hypersaline Environments?

One of the most compelling arguments against a great ecological advantage of the production of gas vesicles is the observation that hypersaline environments (natural salt lakes, saltern evaporation and crystallizer ponds, *etc.*) are seldom dominated by gas-vacuolate types of Archaea. The only exception appears to be *Haloquadratum*, which is often found in very high numbers, as discussed below. It must, however, be realized that culture-dependent approaches in which colonies developing on agar plates are examined are problematic due to the low recovery percentage of colonies as compared to the microscopically observed numbers. Microscopical examination of brines after concentration of the cells by centrifugation is also not effective, as high-speed centrifugation causes collapse of the vesicles due to the high pressure applied to them. It is therefore possible that gas-vacuolate types are more abundant that generally realized, but data are lacking. It would be interesting to examine the abundance at which *gvp* genes turn up in the metagenomes of different hypersaline environments.

*Hbt. salinarum* and *Hfx. mediterranei*, the two species used in most experiments on archaeal gas vesicles, are not at all abundant in aquatic hypersaline environments. Gas-vacuolate *Halobacterium* strains were generally isolated not from salt lakes but from salted fish and salted hides [1,2,3,7,54], and the genus *Halobacterium* contributes very little to the prokaryote community in saltern ponds and salt lakes. *Halobacterium* strains can be specifically enriched and isolated from salterns based on their ability to grow anaerobically on arginine (see above), but their numbers appear to be small [36]. *Halobacterium* 16S rRNA gene sequences seldom turn up in metagenomes and in 16S rRNA gene libraries prepared from such environments. Moreover, the *Halobacterium*-specific glycolipids (sulfated triglycosyl and tetraglycosyl diphytanyl diether lipids) form only a very small fraction, if they are detectable at all, of the total glycolipids in the community [55].

*Hfx. mediterranei* was never shown to be present in high numbers in any environment, in spite of its extremely high versatility: it can grow in a wide range of salt concentrations, use many more substrates for growth than most other halophilic Archaea, can digest a range of polymeric compounds, has a very high growth rate, and excretes halocins—protein antibiotics that inhibit growth of many other members of the *Halobacteriaceae* [1,20].

*Haloplanus* strains were only occasionally isolated, and it is not clear how widespread this genus might be. *Haloplanus*-related 16S rRNA gene sequences were retrieved from an oilfield and from a gypsum crust in a solar saltern [22]. They were also found, albeit at a low frequency, in the metagenome of the Dead Sea in 2007, at a time community densities in the lake were very low [56]. At the time the Dead Sea was subject to deep mixing, so that presence of intact gas vesicles could not be expected. We also know little about the worldwide abundance of gas-vacuolate *Halogeometricum*.

The only extreme halophile with gas vesicles that appears to contribute significantly to the prokaryote community in hypersaline lakes, both natural and artificial, is the square *Hqr. walsbyi*. Its characteristic flat square cells are abundantly found in saltern crystallizer ponds worldwide. First found to be present in numbers as high as 7 × 10^7^ cells per mL of brine in a coastal salt pool on the Sinai peninsula, Egypt, it was since encountered in salterns in Spain, Israel, Mexico, USA, Australia, and elsewhere, sometimes contributing more than half of the prokaryote numbers [57,58]; they exist in certain natural salt lakes as well [59].

### 5.2. Are Natural Communities of Halophilic Archaea Ever Oxygen-Limited?

From the time of the early studies by Petter it was often assumed that the main function of gas vesicles in the *Halobacteriaceae* may be to obtain access to oxygen, potentially a limiting factor in natural brines. Indeed, the community densities of halophilic Archaea and other microorganisms in hypersaline brines in nature are often high (densities of 10^7^–10^8^ cells/mL are not exceptional), and the solubility of oxygen is reduced as salinity increases. 

There have, however, only been very few measurements of actual oxygen concentrations of natural brines and of community respiration rates in such brines. Using a method based on the Winkler titration, Rodriguez-Valera *et al.* [60] measured 0.3–0.8 mg/L (9–25 μM) oxygen in salt-saturated saltern ponds in Spain. Similar values (~20–27 μM) were estimated for crystallizer brine in the Eilat, Israel salterns [61]. Such concentrations are probably not limiting to the community of aerobic halophilic microorganisms in any way. When the Eilat brine, containing 3.9 × 10^7 ^prokaryote cells per mL, most of which were of the *Haloquadratum* type, was incubated in the dark at 30 °C in a closed containers, it took as much as 50 h before oxygen was completely depleted [61]; later experiments in which the oxygen uptake in Eilat crystallizer brine (3 × 10^7^ prokaryote cells per mL) was monitored at 35 °C by an oxygen optode showed oxygen depletion after 32–38 h (R. Pinhassi, E. Maimon, R. Horwitz and A. Oren, unpublished results). In the natural system oxygen is continuously supplied by diffusion from the air and mixing by waves, as well as by photosynthesis by the unicellular halophilic alga *Dunaliella salina* during daytime. Experiments in which the time course of oxygen depletion by cultures of *Hbt. salinarum*, *Hqr. walsbyi* and by Eilat crystallizer brine was followed by means of a Clark-type oxygen microelectrode showed a linear decrease in O_2_ concentrations down to values below 1 μM (M. Krause, G. Panasia, N. Meyer, and A. Oren, unpublished results). This shows that the affinity of halophilic Archaea for oxygen is sufficiently high so that oxygen cannot be expected ever to become limiting in the shallow crystallizer ponds in spite of the presence of the dense microbial communities. Therefore the cells under these situations do not need buoyancy by gas vesicles to obtain oxygen.

### 5.3. Studies on Haloquadratum in Coastal Brine Pools, Sinai Peninsula

The flat, square *Haloquadratum*-type microorganisms were first recognized by Walsby in brine pools on the Red Sea coast at the southern end of the Sinai Peninsula, Egypt [28,35]. Until the organism was brought in culture in 2004 [25,29,30,31], most studies on these fascinating organisms were based on samples collected from these pools. The early reports mentioned positive buoyancy of the square cells, which often were present in pairs, groups of four, eight, sixteen [28,62]; in one case even an assembly of sixty-four cells was observed “like postage stamps in a sheet” [63]. Cells were reported to have accumulated in large numbers on the surface of the brine pool, buoyed up by their gas vesicles [63]. For further studies, gas-vacuolate cells were concentrated by leaving brine samples standing for a few days, collecting cells from the surface, followed by further concentration by low speed centrifugation (accelerated flotation), taking care not to exceed the critical pressure at which gas vesicles start collapsing [28,63,64]. In other microscopy and electron microscopy studies high-speed centrifugation was employed to concentrate the cells, leading to collapse of the gas vesicles [62]. A definite reduction in gas vesicle numbers per cell was seen after application of 0.15 MPa, most of the vesicles had disappeared by 0.25 MPa, and none remained at pressures beyond 0.3 MPa [28].

### 5.4. Studies on Haloquadratum in Saxkoye Lake, Ukraine

Flat square gas-vacuolate cells were collected from the surface waters of the hypersaline Saxkoye Lake, Ukraine [59]. In this study, performed shortly after Walsby had discovered this type of cells in the Sinai brine pool, the nature of the structures found was misinterpreted as square microcolonies, the gas vesicles erroneously considered to be the cells. Although details are lacking, the published information makes it understood that the cells were collected from the surface water film by floating electron microscope grids on top of the brine. Whether indeed these square structures were present at a higher density at the brine surface than in the deeper waters was not reported.

### 5.5. Studies on Haloquadratum in the Crystallizer Ponds of the Eilat Salterns

In the saltern crystallizer ponds of Eilat, square gas-vacuolate cells of the *Hqr. walsbyi* type consistently make up a high percentage of the microbial community; at least 70%–80% of all cells show this characteristic morphology (see also Figure 2). This fact enabled us to obtain information on the polar lipid composition of *Haloquadratum* long before the organism had been brought into culture [58].

The presence of these dense populations in saltern crystallizer ponds presented a unique opportunity to study the possible role of the gas vesicles in the life of *Haloquadratum*. No indications for positive buoyancy were obtained [65]:

(1) When samples of brine were placed in a Petroff-Hauser counting chamber and left to stand for up to 4 hours, cells had not accumulated near the cover slip, but remained distributed evenly within the 20 μM thick space between slide and cover slip. However, when the brine sample was first subjected to pressurization causing collapse of all gas vesicles, most cells sank to the bottom.

(2) When the brine was incubated for up to 5 days at room temperature in diffuse daylight in 1 liter glass cylinders, little change in the vertical distribution of the cells was demonstrated, except for a tendency for a small decrease of cell numbers in the upper layer toward the end of the incubation period. To prevent convection currents to cause mixing of the brine, the experiment was repeated by establishing a salinity gradient from 100% brine below to 90% brine – 10% distilled water on top. The result was similar. 

(3) In “accelerated flotation” experiments in which brine samples were centrifuged at speeds (26 x *g*) for periods of up to 12 h, the cells were still homogeneously distributed throughout the tubes. A similar result was obtained following 60 h centrifugation in a swing-out rotor at 39.1 x *g*. In all these cases the calculated maximum pressure exerted on the cells did not exceed 0.014 MPa, a pressure insufficient to cause collapse of even the weakest gas vesicles. When the brine had earlier been subjected to pressure above 0.2 MPa and all gas vesicles had been collapsed, cell densities at the lower end of the centrifuge tube were significantly higher than at near the surface after 60 h of low speed centrifugation.

These observations suggest that the gas vesicles present in the square halophilic Archaea in the saltern ponds of Eilat provide negligible floating/sinking velocity to the cells. The gas vesicles were sufficient to provide neutral buoyancy, but not positive buoyancy that may allow the cells to float under optimal conditions. In this respect the properties of the *Haloquadratum* community in the Eilat salterns may have differed from that in the Sinai brine pool. If the gas-vacuolate flat square cells do not float in a test tube in the laboratory, they cannot be expected to buoy up in the natural environment where wind, waves, and water currents will tend to disperse them equally at all depths [65].

## 6. Cell Size and Colony Size as Critical Parameters Affecting Buoyancy of Gas-Vacuolate Prokaryotes

The rate at which a particle rises or sinks in a liquid medium depends not only on the difference in density between the particle and the solution and on the viscosity of the solution (which is about twice as high in saturated brines than in freshwater), but also on the size of the particle. When estimating the rate of sinking or flotation of a prokaryotic cell in a water column, the first approximation is that determined by Stokes's equation for a spherical particle [66]:

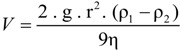

where V is the velocity of fall, g is the acceleration of gravity, r is the 'equivalent' radius of the particle, ρ_l_ is the density of particle, ρ_2_ is the density of medium, and η is the dynamic viscosity of medium. For a non-spherical particle such as a flat *Haloquadratum* cell certain corrections must be made, as the hydrodynamic behavior of the square cells cannot be simulated by a sphere. A flat particle will encounter increased drag forces compared to a spherical particle of the same volume when moving in the water column with the direction of motion parallel to the short axis of that particle. For the flat square cells, the shape increases the effective Stokes radius approximately two-fold [65,67].

Based on different assumptions to estimate the density of *Haloquadratum* cells, flotation rates calculated from Stokes's equation were in the order of a few millimeters per day only. Thus, even when the water column is not subjected to any mixing by waves and currents, the square Archaea in the salterns will not float toward the surface of the brine at a significant speed. Other gas-vacuolate members of the *Halobacteriaceae* such as *Hrr. vacuolatum* and *Hfx. mediterranei* also have cells smaller than 2–3 mM [1,20,26], and therefore they cannot be expected to float at higher speeds.

Gas vesicle-containing cyanobacteria such as *Microcystis*, *Anabaena*, and *Trichodesmium* can form surface blooms because their cells are largeand their content of gas vesicles can be very high, causing a large difference in density between the cells and their medium. Furthermore, they generally grow in colonies, filaments or bundles of filaments, thus increasing their effective radius in Stokes's equation, and they live in a medium of half the viscosity of that of the halophilic Archaea. Increasing the effective radius, resulting in higher flotation rates, may be an option for *Haloquadratum*, which is known to form small sheets when cells do not separate after division. The material from the Sinai brine pool showed an abundance of sheets of four to eight cells [28,52,63,64], and the observation of a sheet of 64 cells [63] suggests that formation of such ‘colonies’ may increase the efficiency of flotation, provided their density is indeed lower than that of the brine.

## 7. Do the Gas Vesicles of *Haloquadratum* Serve to Optimize Light Absorption?

Another hypothesis to explain the possible function of gas vesicles in *Haloquadratum*, first proposed by Bolhuis *et al.* [33], is based on the observation that the gas vesicles are often mainly located close to the cell periphery. Photographs of cells with most or all gas vesicles close to the edges are found in many publications [28,31,64]. Sheets are so thin that they bulge slightly with gas vesicles along their edges. Light absorption by thin sheets can be highly efficient, and especially by sheets oriented normal to the incoming light. It was postulated that the arrangement of the gas vesicles may aid the cells to position themselves parallel to the water surface. Such a horizontal positioning would aid the cells in collecting as many photons as possible to be absorbed by the bacteriorhodopsin proton pump, present in *Haloquadratum*, to generate ATP. The fact that *Haloquadratum* lacks flagella may be important here: flagellar movement would have caused the cells to rotate [35]. A similar location of peripheral gas vesicles in larger cells was found in *Hpn. natans* (Figure 1, left panel), an organism that also does not show active motility.

**Figure 3:**
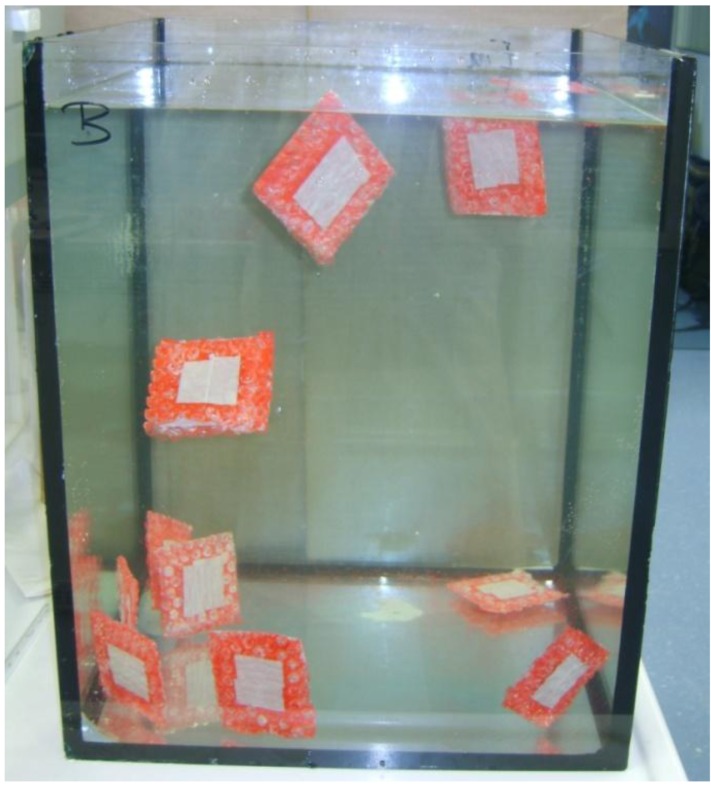
Random orientation of model *Haloquadratum*-type “Archaea” with peripheral “gas vesicles” suspended in water. The model“cells” were crafted from thin glass plates (10 cm diameter) glass with bubble wrap at both sides along the edges to provide near-neutral buoyancy. With thanks to the staff of the Max Planck Institute for Marine Microbiology, Bremen, for the facilities provided.

One possible problem with this hypothesis is the fact that the salt lakes and shallow pools where organisms such as *Haloquadratum* thrive are generally exposed to very high light intensities, so that light is not likely to become a limiting factor. The opposite may be true: the cells have different mechanisms to protect themselves against damage by high levels of radiation. Another question to be asked is whether the geometry of the cells and the spatial arrangement of the gas vesicles indeed allow the cells to position themselves parallel to the water surface and so maximize light absorption. The small cells, with their accordingly low Reynolds number [66,68] experience their medium as extremely viscous, and will not easily rotate; Brownian motion will further tend to randomize the cells' orientation. Moreover, in a macroscopic model in which much larger square “cells” (with an accordingly higher Reynolds number) with near-neutral buoyancy provided by peripheral “gas vesicles”, the cells remained oriented randomly in the water rather than positioning themselves horizontally (Figure 3). 

Positioning of the square cells to maximize light exposure can probably be expected only if the cells are sufficiently flexible so that the margins with the gas vesicles will bend upward and the cells become somewhat cup-shaped. Indeed the flat cells show a degree of flexibility as shown by photographs of exceptionally large “folded” cells [29,30]. It remains to be ascertained whether ‘standard’ small square cells with a diameter of 2–3 μM are also flexible enough to become deformed by the buoyancy of the gas vesicles.

## 8. Do Gas Vesicles Serve to Increase the Cell Surface/Cytoplasmic Volume Ratio?

An altogether different idea why some halophilic Archaea may possess gas vesicles, a hypothesis that has nothing to do with cell buoyancy, oxygen limitation and light harvesting, was proposed already in the 1950s by Houwink [6] in his electron microscope study of *Hbt. salinarum*: “Since the gas vacuoles fill part of the space within the cell wall, the ratio of volume of the cytoplasm/surface area of the cell wall is smaller than it would be if the gas vacuoles were lacking.” And he added: “It is quite another question whether a large surface area with respect to the volume of the cytoplasm is profitable to the organism. There was found no indication that this was the case with *H. halobium*” (now: *Hbt. salinarum*).

This hypothesis was revived in several recent studies. It was argued that a larger surface-to volume-ratio, resulting in shorter diffusion times, may be important for especially for organisms growing at low temperatures [18]. Gas vesicle production leads to a large surface area of the cell for nutrient acquisition but a relatively small volume of the cytoplasm [8,18,43]. A low growth temperature is one of the factors inducing gas vesicle synthesis in *Halobacterium* [47,69]. This may explain why the gas-vacuolate *Halobacterium* strains NRC-1 and PHH1 grow faster in the cold compared to the gas vesicle-negative strain PHH4 [48].

## 9. The Function of Gas Vesicles in the Life of Endospore-Forming Anaerobic Bacteria in the Sediments of Hypersaline Lakes

Even more enigmatic than the function of gas vesicles in the aerobic halophilic Archaea is the finding of gas vesicles in two obligate anaerobic halophilic representatives of the domain Bacteria (*Firmicutes*, Order *Halanaerobiales*): *Sporohalobacter lortetii* (basonym: *Clostridium lortetii*) [70,71] and *Orenia sivashensis* [72]. These were isolated from Dead Sea sediment and from sediment of Lake Sivash, Crimea, respectively.

Vegetative cells of *S. lortetii* do not carry gas vesicles. These structures are synthesized concomitant with the production of endospores, and they remain attached to the mature spores. Also in *O. sivashensis* gas vesicles were found in the mature spores, located between the inner membrane of the exosporium and the spore coat, but also in different parts of the vegetative cells. Similar anaerobes that produce endospores with attached gas vesicles are known from soils and other non-hypersaline environments. It was postulated that such spores, which are oxygen-resistant, may rise to the water surface and become dispersed by water currents until reaching a new anaerobic environment suitable for germination [73]. However, to what extent this mechanism may indeed function is unknown. Endospores of *S. lortetii* and *O. sivashensis* were never shown to float. Moreover, *S. lortetii* was recovered from Dead Sea sediment at a depth of 60 m, where the hydrostatic pressure far exceeds the critical collapse pressure of the gas vesicles, so that functional gas vesicles cannot exist at the site.

## 10. Epilogue

Helge Larsen, who in 1967 wrote his pioneering paper on the nature of the gas vesicles of *Halobacterium* [7], delivered in 1972 a lecture entitled “The Halobacteria's Confusion to Biology” [74], a title paraphrasing Kluyver and van Niel's “The Microbe's Contribution to Biology”. Now, forty years later, considerable confusion still exists about the possible function of the gas vesicles in the life of *Halobacterium* and its gas-vacuolate relatives among the *Halobacteriaceae*. Whatever the ecological advantage of the production of gas vesicles may be, offsetting the cost of their production is as yet unclear.

We have learned very much about the structure of gas vesicles, the genes involved in their formation, and the regulation of their production in two model species: strains of *Hbt. salinarum* and *Hfx. mediterranei*. All those studies, however, have not yet led to an unequivocal answer why some members of the family need gas vesicles to remain competitive in their natural environment. A *Haloquadratum* culture left standing on the bench does not contain cells at the bottom; rather, the cells will stay in the water column, but will not float to the surface. Thriving at the surface of lakes and ponds would be very harsh: exposure to UV light, even more dryness, and if rainfall comes the cells will disrupt and die. Also, most isolates of *Hbt. salinarum* are unable to float; only the laboratory strains PHH1 and NRC-1 float at the surface when the culture is left standing on the bench. The *c-*vac expressing strains SB3, GN101, and PHH4 will remain distributed in the water column. This may show that the ‘overproduction’ of gas vesicles by PHH1 and NRC-1 might be a laboratory artifact. Still, the fact that the property is maintained in nature, in spite of the obvious cost to the cell that produces the vesicles, proves that there must be some benefits involved. In conclusion, the ecological advantage of gas vesicle production is not yet understood.

Kessel *et al.*, in a 1985 paper on the square gas-vacuolate microorganisms from the Sinai brine pools [52], wrote: “Although information is accumulating on the physiology and chemistry of gas vesicles in halobacteria, we still need experimental confirmation of their ecological significance in these organisms”, and: “Laboratory experiments… will at best only test whether it is *feasible* for the gas vacuole to provide benefits through buoyancy. The actual benefits can only be assessed by making observations and performing experiments in the field”. These words remain very true indeed, more than a quarter of century later.
